# Heat shock protein A1 inhibits the replication of foot-and-mouth disease virus by degrading viral RNA polymerase 3D through chaperone-mediated autophagy

**DOI:** 10.1128/jvi.00168-25

**Published:** 2025-03-31

**Authors:** Mei Ren, Haiqian Zhou, Jin-en Wu, Jia-ning Wang, Xuefei Wang, Sahibzada Waheed Abdullah, Huichen Guo, Shiqi Sun

**Affiliations:** 1 State Key Laboratory for Animal Disease Control and Prevention, College of Veterinary Medicine, Lanzhou University, Lanzhou Veterinary Research Institute, Chinese Academy of Agricultural Sciences 111658 , Lanzhou, China; 2 Gembloux Agro-Biotech, University of Liege 82209, Gembloux, Belgium; 3 Livestock and Dairy Development Department Peshawar, Government of Khyber Pakhtunkhwa 364971, Peshawar, Pakistan; University of Kentucky College of Medicine, Lexington, Kentucky, USA

**Keywords:** foot-and-mouth disease virus, heat shock protein, HSPA1, RNA dependent RNA polymerase, 3D polymerase, chaperone mediated autophagy

## Abstract

Foot-and-mouth disease virus (FMDV), a member of the *Picornaviridae* family, is a single-stranded, positive-sense RNA virus. Heat shock protein A1 (HSPA1) has been shown to influence the entry, translation, assembly, and release of enterovirus A71 (EV-A71), another *Picornaviridae* family member. In this study, we demonstrate that HSPA1 plays a different role in the replication of FMDV. By investigating various stages of virus replication, we found that HSPA1 specifically inhibits the RNA replication stage in which HSPA1 inhibits viral RNA replication by degrading the viral RNA-dependent RNA polymerase (RdRp), 3D protein. In the presence of specific inhibitors, we find out that this degradation occurs through the autophagy pathway. Activation and blockage of chaperone-mediated autophagy (CMA) demonstrate that HSPA1 degrades 3D through the CMA pathway. Mutation analysis reveals that _421_QEKLI_425_ is the key motif in 3D responsible for HSPA1-mediated CMA degradation. In summary, this study shows that HSPA1 can degrade the viral 3D protein through the CMA pathway, thereby inhibiting the RNA replication of FMDV and interfering with virus infection. This study, for the first time, demonstrates that HSPA1 employs its chaperone function to mediate the degradation of the FMDV RdRp, revealing the crucial role of HSPA1 in the FMDV infection process and suggesting that HSPA1 could be a potential target for the prevention and treatment of FMDV infection.

**IMPORTANCE:**

Viral RNA replication is the key stage in understanding the pathogenic mechanisms of foot-and-mouth disease virus (FMDV). During this process, the viral non-structural protein 3D serves as an RNA-dependent RNA polymerase (RdRp) to synthesize progeny RNA using the viral genomic RNA as a template. However, the regulatory effect of host cells on FMDV 3D proteins has not yet been studied. In this study, we find that heat shock protein A1 (HSPA1) degrades the viral 3D protein through the chaperone-mediated autophagy (CMA) pathway, thereby inhibiting the RNA replication of FMDV and interfering with virus infection. This study, for the first time, demonstrates that HSPA1 employs its chaperone function to mediate the degradation of the FMDV RdRp.

## INTRODUCTION

Foot-and-mouth disease virus (FMDV) is a member of the *Picornaviridae* family, possessing a single-stranded, positive-sense RNA genome encased in a capsid composed of viral structural proteins. FMDV causes foot-and-mouth disease (FMD), an acute, febrile, and highly contagious disease that affects cloven-hoofed animals such as pigs, cows, and sheep (1, 2). FMD has been classified by the World Organization for Animal Health (WOAH, formerly known as OIE) as a notifiable disease, requiring member countries to report any occurrences of FMD within their territories to WOAH (3). This virus can be transmitted through respiratory secretions and excretions such as blisters, saliva, and milk (4, 5). While adult animals generally experience mild symptoms, juvenile animals often suffer high mortality rates. Consequently, FMDV poses a serious threat to the livestock industry and international trade in animals and animal products. Given the severe impact of FMD, efforts to eradicate and control the virus are crucial. To develop effective prevention and control strategies, extensive research has focused on understanding the pathogenic mechanisms of FMDV at various stages of its infection cycle.

RNA replication is the key factor in understanding the pathogenic mechanisms of FMDV. Once inside the host cell, the virus dissociates, releasing its genome and initiating RNA replication (6). RNA replication occurs in the cytoplasm. Firstly, the viral positive-sense RNA is translated into viral proteins. The viral genome, approximately 8.3 kb in length, is a single-stranded positive-sense RNA that includes one large open reading frame flanked by two untranslated regions (UTRs) at both ends. The viral genome encodes four structural proteins (VP1, VP2, VP3, and VP4) and eight non-structural proteins (L, 2A, 2B, 2C, 3A, 3B, 3C, and 3D) that regulate RNA replication, protein folding, and virus assembly (7). The non-structural protein 3D, an RNA-dependent RNA polymerase (RdRp), synthesizes negative-sense RNA using the viral genomic RNA as a template within the replication complex (RC). This negative-sense RNA is then used to produce more positive-sense RNA (8). During this process, a large quantity of viral RNA is generated to support the translation of viral proteins and the formation of new viral particles. These newly synthesized viral proteins gradually accumulate within the cell and, along with newly synthesized viral RNA, assemble into new viral particles. These newly synthesized viral particles ultimately exit the infected cells and spread to the surrounding environment.

Heat shock proteins (HSPs) are a class of stress-induced proteins that are ubiquitously present in prokaryotes and eukaryotes. Based on the molecular weight and amino acid (AA) sequence similarity, HSPs are classified into five families: small HSP (HSPB), HSP70 (HSPA), HSP40 (DNAJ), HSP90 (HSPC), and HSP110 (HSPH) (9). The high evolutionary conservation of the HSPA family suggests their universally important physiological functions. In picornavirus infections, the HSPs exhibit diverse and distinct roles depending on the virus (10–16). The reduction of HSP90β decreased the correct assembly of enterovirus A71 (EV-A71) particles (13). HSP60 plays a role in FMDV infection by regulating viral replication and participating in viral morphogenesis (17). Additionally, the HSPA family (HSPAs) has been reported to regulate all phases of the EV-A71 life cycle (16). HSPA1, also known as HSP70.1 or HSP72, a member of HSPAs, facilitates EV-A71 entry and translation while also contributing to viral particle assembly and release. The inhibitor of HSPAs not only blocks the replication of EV-A71 but also inhibits the replication of other picornaviruses, such as coxsackievirus (CV) A16, CVB1, CVB3, and echovirus 11. However, the role of HSPA1 in the life cycle of FMDV remains unclear.

In this study, we investigate the role of HSPA1 in FMDV infection. As a molecular chaperone protein, it facilitates the correct folding of newly translated and misfolded proteins and stabilizes or degrades mutated proteins (18). Through modulating the expression of HSPA1, we find out that HSPA1 inhibits FMDV infection, particularly during the RNA replication stage of the virus. After further exploration, we confirmed that HSPA1 degrades the viral RdRp protein 3D through the chaperone-mediated autophagy (CMA) pathway with the key motif on the 3D protein, _421_QEKLI_425_. This study is the first to demonstrate that HSPA1 utilizes its chaperone function to mediate the degradation of FMDV RdRp.

## MATERIALS AND METHODS

### Cells and viruses

PK-15 (porcine kidney; ATCC CCL-33) cells and BHK-21 (baby hamster kidney; ATCC CCL-10) cells were obtained from Lanzhou veterinary research institute and cultured in Dulbecco’s Modified Eagle’s Medium (Gibco, California, USA) supplemented with 10% fetal bovine serum (FBS; Gibco), penicillin (100 U/mL), and streptomycin (100 µg/mL) (Gibco) at 37°C in a humidified atmosphere containing 5% CO_2_.

FMDV serotype O strain O/China/99 (GenBank accession no. AF506822.2) was obtained from the WOAH/China National Foot-and-Mouth Disease Reference Laboratory (Lanzhou, China). FMDV was propagated in BHK-21 cells, and the viral titers were determined using a 50% tissue culture infective dose (TCID_50_) assay in BHK-21 cells.

### Antibodies and reagents

Mouse anti-HSPA1 monoclonal antibody (mAb) (66183-1-Ig), anti-DDK (66008-4-Ig) mAb, anti-HSC70 mAb (66442-1-Ig), rabbit anti-HA polyclonal antibody (pAb) (51064-2-AP), anti-LC3 pAb (14600-1-AP), and anti-LAMP2 pAb (27823-1-AP) were purchased from Proteintech (Wuhan, China). Mouse anti-β-actin mAb was purchased from CWBIO (Beijing, China). Rabbit anti-Integrin α5β6 mAb was purchased from Beyotime (Shanghai, China). Pig anti-3D mAb was kindly provided by Dr. Chao Shen from Wuhan University. Pig serum pAb against FMDV was prepared in our laboratory. Protein G Sepharose 4 Fast Flow was purchased from Cytiva (Uppsala, Sweden). Lipofectamine RNAiMAX and Lipofectamine 2000 were purchased from Invitrogen (California, USA). Cycloheximide (CHX), MG132, chloroquine (CQ), Z-VAD-FMK, CA77.1, and Apoptozole were purchased from MedChemExpress (Monmouth Junction, NJ). All drugs in this study were dissolved in 0.1% DMSO. Tragacanth gum powder was purchased from Wako Pure Chemical Industries (Osaka, Japan).

### RNA interference

For RNA interference (RNAi), small interfering RNAs (siRNAs) targeting candidate genes and negative-control (NC) siRNA were synthesized by Genepharma company (Shanghai, China). The sequences of the siRNAs for Sus scrofa HSPA1 are as follows: si1629, 5′-CGUACGCCUUCAACAUGAATT-3′; si1274, 5′-CAGAUCUUCACCACGUACUTT-3′; si304, 5′-AAGGUGCAGGUGAGCUACATT-3′; and si502, 5′-AACGUGCUGCGGAUCAUCATT-3′. The sequences of the siRNAs for *Sus scrofa* LAMP2A are as follows: si966, 5′-GCGGCAGCAACAUCAAAUATT-3′; si1045, 5′-CAGCGUGUAUUUGGUUAAUTT-3′; si276, 5′-GGCAGAUGAAUUUCACAAUTT-3′; and si451, 5′-GGAAUCGUCUAGUUAUUUATT-3′. The siRNA transfection was conducted using Lipofectamine RNAiMAX when cell density was approximately 80%, according to the manufacturer’s instructions.

### Plasmid constructs

Mammalian expression plasmids for FMDV non-structural protein L, 2B, 2C, 3A, 3BCD, 3C, 3D, and the dual-luciferase plasmid psiCHECK-FMDV were previously synthesized in our laboratory (19, 20). The HA-tagged HSPA1 plasmid was constructed using the pCMV-HA-C vector available in our laboratory (21). The coding sequence of HSPA1 was amplified from PK-15 cells, with *Sal* I and *Kpn* I restriction sites added at 5′ and 3′ ends, respectively, using HSPA1 primers (Table 1). This sequence was inserted into the vector through digestion and ligation, resulting in the overexpression plasmid HA-tagged HSPA1 (HA-HSPA1). Plasmids with HSPA1 truncated version were constructed based on the HA-HSPA1 with primers listed in Table 1 (HSPA1-1-385 and HSPA1-386-640). The mutated Flag-3D plasmids were constructed through site-directed mutagenesis PCR, using the original Flag-3D plasmid as a template and the primers listed in Table 1. The plasmid pCMV-mCherry-GFP-LC3B was purchased from Beyotime (Shanghai, China). All primers were synthesized by Tsingke (Beijing, China). All DNA constructs were confirmed by sequencing.

**TABLE 1:** List of primers

Target	Sequence (5′−3′)
HSPA1	F: CGCGTCGACGCCACCATGGCGAAGAGCGTGGC R: GGGGTACCATCCACCTCCTCGATGGTGGGGCC
HSPA1-1-385	F: CGCGTCGACGCCACCATGGCGAAGAGCGTGGC R: GGGGTACCCGACTTGTCGCCCATCAGGATGGC
HSPA1-386-640	F: CGCGTCGACGCCACCATGGAGAACGTGCAGGA R: GGGGTACCATCCACCTCCTCGATGGTGGGGCC
3D-ATFLK	F: AGAATACAAGTTTGCCTGTGCGACCTTCCTGAAGGACGAA R: TTCGTCCTTCAGGAAGGTCGCACAGGCAAACTTGTATTCT
3D-QAFLK	F: CAAGTTTGCCTGTCAGGCCTTCCTGAAGGACGA R: AAGTTCGTCTGCCAGGCCTTCCTGAAGGACG
3D-QTALK	F: CAAGTTTGCCTGTCAGACCGCCCTGAAGGACGAAATTCGC R: GCGAATTTCGTCCTTCAGGGCGGTCTGACAGGCAAACTTG
3D-QTFAK	F: TTGCCTGTCAGACCTTCGCGAAGGACGAAATTCGCC R: GGCGAATTTCGTCCTTCGCGAAGGTCTGACAGGCAA
3D-QTFLA	F: CTGTCAGACCTTCCTGGCGGACGAAATTCGCCCG R: CGGGCGAATTTCGTCCGCCAGGAAGGTCTGACAG
3D-AEKLI	F: GCACGCCGTGGGACCATAGCGGAGAAGTTGATCT R: AGATCAACTTCTCCGCTATGGTCCCACGGCGTGC
3D-QAKLI	F: CGTGGGACCATACAGGCGAAGTTGATCTCCGTG R: CACGGAGATCAACTTCGCCTGTATGGTCCCACG
3D-QEALI	F: GTGGGACCATACAGGAGGCGTTGATCTCCGTGGCAG R: CTGCCACGGAGATCAACGCCTCCTGTATGGTCCCAC
3D-QEKAI	F: GACCATACAGGAGAAGGCGATCTCCGTGGCAGGG R: CCCTGCCACGGAGATCGCCTTCTCCTGTATGGTC
3D-QEKLA	F: CATACAGGAGAAGTTGGCCTCCGTGGCAGGGCTC R: GAGCCCTGCCACGGAGGCCAACTTCTCCTGTATG
FMDV	F: CAAACCTGTGATGGCTTCGA R: CCGGTACTCGTCAGGTCCA
*Sus scrofa* GAPDH	F: ACATGGCCTCCAAGGAGTAAGA R: GATCGAGTTGGGGCTGTGACT

### Cell viability assay

Cells were incubated with indicated drugs for 24 hours or transfected with specific siRNAs or plasmids for 48 or 24 hours, respectively, at approximately 60% confluence in 96-well plates. Cell viability was assessed using the MTT assay. For this method, 10 µL of CellTiter 96 AQueous One Solution Cell Proliferation Assay reagent (Promega, WI, USA) was added directly to the cells, which were then incubated for 4 hours. The absorbance was measured at 490 nm using a Synergy H1 microplate Reader (Biotek, USA).

### RNA extraction and quantitative real-time PCR

Total RNA was extracted from cells using RNAi Plus (TaKaRa) according to the manufacturer’s protocol. Reverse transcription was then performed using a 5× RT master mix (TaKaRa) to synthesize cDNA. Transcript levels of FMDV and GAPDH were measured through quantitative real-time PCR (RT-qPCR) using primers listed in Table 1. For the detection of viral negative-sense RNA, reverse transcription was carried out using only the forward primer from the FMDV primer set, resulting in a single cDNA sequence representing the viral negative-sense RNA (22).

### Virus titration

Virus infectivity was quantified using endpoint dilution assays. Serial dilution samples of virus were used to infect designated cells in 96-well plates. The tissue culture infectious dose 50 (TCID_50_) was then calculated employing the Reed-Muench method.

### Immunoprecipitation assay

PK-15 cells were lysed using radioimmunoprecipitation assay (RIPA) buffer (Beyotime Biotechnology, Shanghai, China) for 1 hour on ice, followed by centrifugation at 15,000 × *g* for 20 minutes at 4°C. The supernatants were then subjected to immunoprecipitation overnight at 4°C using the appropriate antibodies. Immune complexes were incubated with Protein G-agarose beads (GE Healthcare, Chicago, IL, USA) for 2 hours, washed six times with lysis buffer, and finally eluted in 1×  SDS-PAGE sample buffer for subsequent immunoblotting analysis.

### Dual-luciferase reporter assay

For the overexpression assays, PK-15 cells were cultured in 12-well plates until reaching 60% confluence, then cotransfected with HA-HSPA1 and psiCHECK-FMDV plasmids using Lipofectamine 2000. After 24 hours of incubation, cells were lysed and samples harvested. For the knockdown assays, cells were transfected with siRNA targeting HSPA1 using RNAiMAX and similarly incubated for 24 hours. Firefly luciferase (FLuc) and Renilla luciferase (RLuc) activities were subsequently measured using a dual-luciferase reporter assay system (Promega), according to the manufacturer’s instructions. The ratio of FLuc to RLuc expression was used to assess the relative activity of the FMDV-5′UTR promoter (23, 24).

### Immunoblotting assay

Cells were lysed using an SDS loading buffer to extract the total protein fraction. The proteins were then denatured at 100°C for 10 minutes, separated via SDS-PAGE, and subsequently transferred to nitrocellulose (NC) membranes. The membranes were blocked for 1 hour using 5% skim milk, incubated overnight with primary antibodies, and washed six times with Tris-buffered saline–Tween (TBST). After this, membranes were incubated for 1 hour with horseradish peroxidase-conjugated secondary antibodies and washed again six times with TBST. The membranes were then treated with an enhanced chemiluminescence detection reagent (Thermo Fisher Scientific, Inc., Rockford, IL, USA) to visualize protein bands.

### Plaque assay

When the cell density reached 90%, serial dilutions of the virus were added to six-well plates. The plates were gently agitated to ensure even coverage of the cells with the virus, then placed in a cell culture incubator to allow the virus to adsorb for 1 hour. During this period, the plates were changed every 10 minutes to prevent drying and ensure even distribution of the virus. Afterward, the virus solution was discarded, and a tragacanth gum culture medium was added to the plates. The plates were then returned to the incubator for an additional 48 hours without disturbance. Finally, the medium was discarded, the cells were washed twice with PBS, fixed with 4% paraformaldehyde, stained with crystal violet for 1 hour, and visually assessed for results.

### Statistical analysis

Data were analyzed using one-way analysis of variance to assess the significance of differences among groups. This method was applied to all experiments, except for the assessment of HSPA1’s impact on FMDV during viral entry, where a student’s *t*-test was employed to compare two groups. Statistical significance was established at *P* < 0.05 for all tests.

## RESULTS

### HSPA1 inhibited FMDV infection

**Fig 1:**
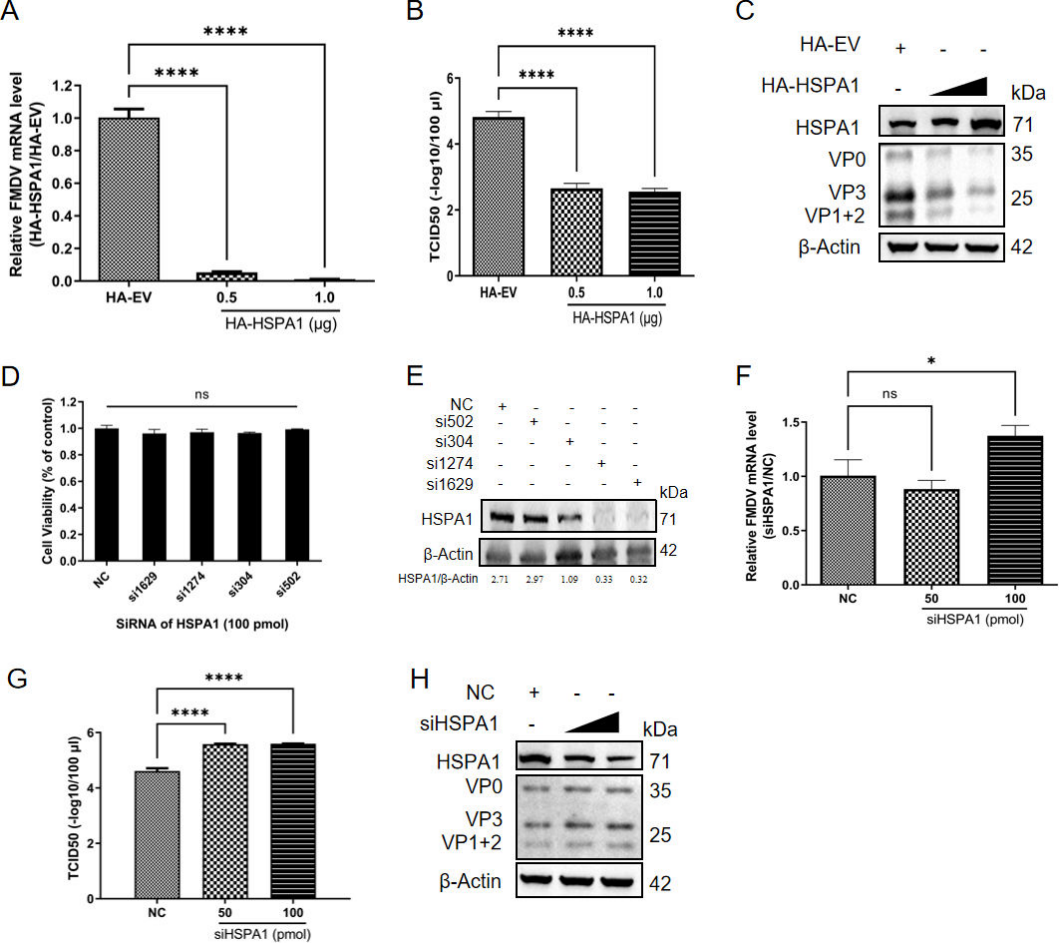
HSPA1 inhibited FMDV infection. PK-15 cells were transfected with an HA-tagged empty plasmid (HA-EV) or a full-length HSPA1 overexpression plasmid (HA-HSPA1). After 24 hours, the cells were infected with FMDV type O at an MOI of 1. Samples were collected at 8 hpi to assess relative FMDV RNA levels (**A**), viral titer (expressed in logarithmic form) (**B**), and viral structural protein level (**C**). (**D**) For the knockdown experiment, the cell viability of HSPA1 siRNA was determined using the MTT assay. (**E**) The knockdown efficiency of siRNA targeting HSPA1 was verified. PK-15 cells were transfected with four groups of siRNAs targeting HSPA1 or NC. After 24 hours, the cells were harvested. Immunoblotting analysis was performed to determine the protein level of HSPA1. After verifying the siRNAs targeting HSPA1, the cells were infected with FMDV type O at an MOI of 1 at 24 hours post-transfection. Samples were collected at 8 hpi to measure relative FMDV RNA levels (**F**), viral titers (expressed in logarithmic form) (**G**), and viral protein levels (**H**). Data were presented as the mean ± standard deviation from three independent experiments. Statistical significance was denoted as follows: *, *P* < 0.05; ****, *P* < 0.0001; ns, not significant.

Considering that the variations in HSP60 protein levels impact FMDV infection and HSPA1’s established role in EV-A71 infection (16, 17), we proposed that changes in HSPA1 protein levels may similarly influence FMDV infection. To explore the role of HSPA1 in FMDV infection, we conducted overexpression and knockdown assays in PK-15 cells. We constructed an HA-tagged HSPA1 overexpression plasmid (HA-HSPA1) using a sequence derived from porcine cells (PK-15 cells) and transfected it, along with an empty vector (HA-EV), into PK-15 cells. Twenty-four hours post-transfection, the cells were infected with FMDV at a multiplicity of infection (MOI) of 1 for another 8 hours. Cells overexpressing HSPA1 exhibited significantly reduced RNA levels and viral titers compared to the HA-EV group, displaying a dose-dependent decrease (Fig. 1A and B). Observation of viral structural protein levels revealed that overexpression of HSPA1 also inhibited virus infection (Fig. 1C). To knock down HSPA1, the cytotoxicity of four siRNAs targeting HSPA1 was evaluated in PK-15 cells using the MTT assay (Fig. 1D), while their knockdown efficiencies were assessed 24 hours post-transfection in PK-15 cells (Fig. 1E). Among these, si1629 was found to be the most effective for the knockdown of HSPA1 in the PK-15 cell line (Fig. 1E), hereafter referred to as siHSPA1. Cells transfected with siHSPA1, or a negative control (NC) for 24 hours were then infected with FMDV at an MOI of 1. Knockdown of HSPA1 resulted in a significant increase in viral RNA levels and titers at 8 hours post-infection (hpi) (Fig. 1F and G). At the protein level, reduced HSPA1 expression promoted virus infection (Fig. 1H). Collectively, these results demonstrated HSPA1’s inhibitory effect on FMDV infection.

### HSPA1 inhibited the RNA replication of FMDV

**Fig 2:**
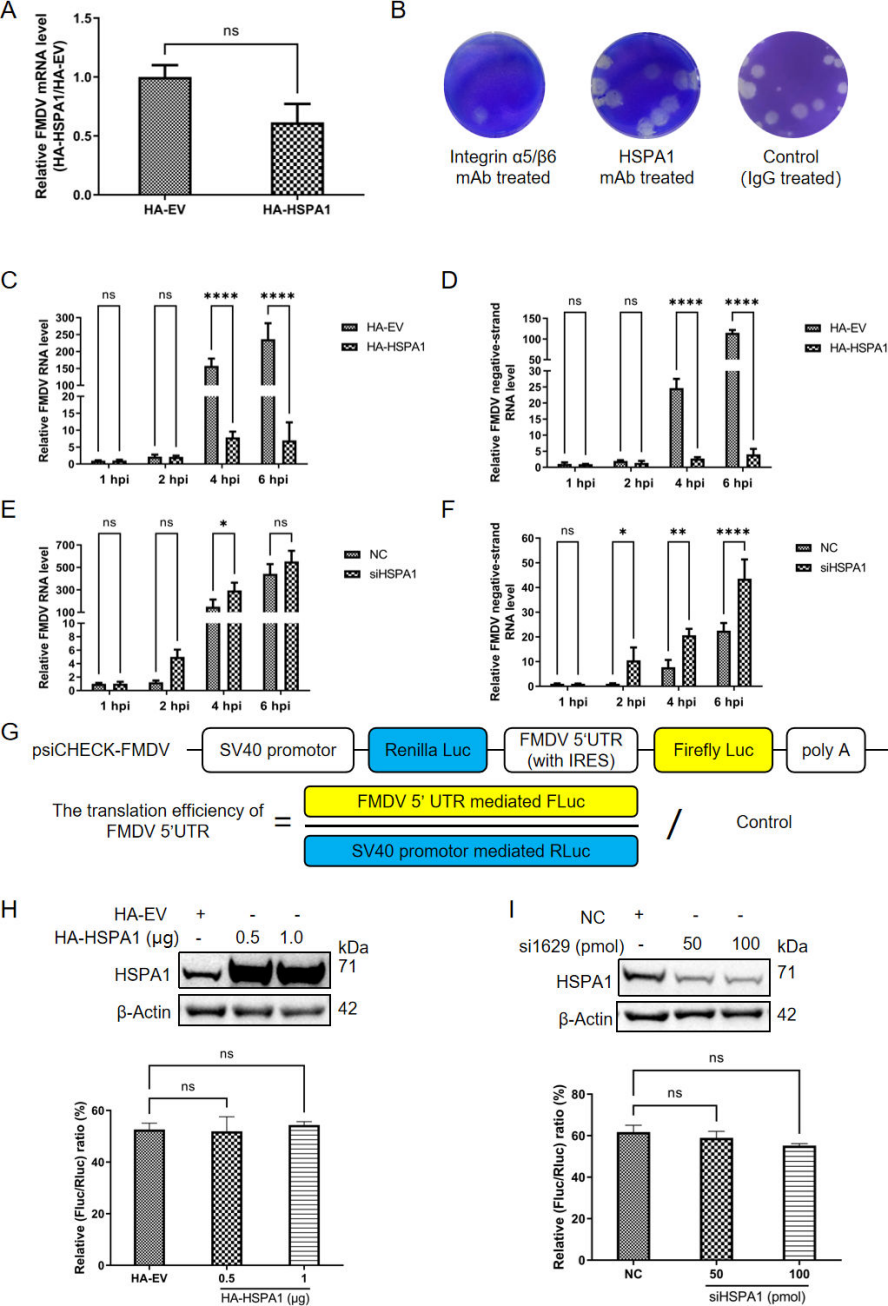
HSPA1 inhibited the RNA replication of FMDV. (**A**) PK-15 cells were transfected with HA-EV or HA-HSPA1. After 24 hours, the cells were infected with FMDV type O at an MOI of 5. Samples were collected at 1 hpi to assess relative FMDV RNA levels. (**B**) When the density of PK-15 cells in the 12-well plate reached 90%, they were incubated at room temperature with antibodies targeting the virus entry receptor integrin α5β6, HSPA1, or IgG, separately. After 1 hour of incubation, the cells were subjected to a plaque assay. The results of the plaque assay were observed after 48 hours. To overexpress HSPA1, PK-15 cells were transfected with either HA-EV or HA-HSPA1, and after 24 hours, infected with FMDV type O at an MOI of 1. Samples were collected at the indicated time points to detect relative FMDV RNA (**C**) and relative FMDV negative-strand RNA (**D**). To knock down HSPA1, PK-15 cells were transfected with either siHSPA1 or NC. After 24 hours of transfection, the cells were infected with FMDV type O at an MOI of 1. Samples were collected at the indicated time points to measure relative FMDV RNA (**E**) and relative FMDV negative-strand RNA (**F**). (**G**) A schematic diagram of the dual-luciferase plasmid psiCHECK-FMDV. (**H**) PK-15 cells were co-transfected with psiCHECK-FMDV and either HA-HSPA1 or HA-EV. After 24 hours, samples were harvested for immunoblotting and dual-luciferase assay analyses. (**I**) For the knockdown experiment, PK-15 cells were transfected with either siHSPA1 or NC. After 24 hours, the cells were transfected with psiCHECK-FMDV, and samples were collected 24 hours post-transfection for immunoblotting and dual-luciferase assay analyses. Data were presented as the mean ± standard deviation from three independent experiments. Statistical significance was indicated as follows: *, *P* < 0.05; **, *P* < 0.01; ****, *P* < 0.0001; ns, not significant.

A previous study showed that HSPA1 was involved in many stages of EV-A71 infection, including its entry, translation, assembly, and release (16). Therefore, we further investigated its role at various stages of FMDV infection. The attachment and entry of FMDV primarily involved cell surface receptors such as integrin receptors and the heparan sulfate (HS) receptor (25–27). HSPA1 was presented not only in the cytoplasm but also on the cell membrane under certain conditions (28). We next considered the possibility that HSPA1 might influence the entry stage of FMDV. To verify this, we overexpressed HSPA1 in PK-15 cells, and after 24 hours, infected the cells with FMDV at an MOI of 5. At 1 hpi, the supernatant was discarded, the cells were washed with PBS, and then collected. The results indicated there is no significant difference in the levels of viral RNA between the HA-HSPA1 and HA-EV groups (Fig. 2A). Additionally, we treated PK15 cells separately with mAb against integrin α5β6, the main cell surface receptor for FMDV entry (25), HSPA1 mAb, and IgG antibody as negative control. A plaque assay was conducted 1 hour later. The results demonstrated that virus entry was blocked in the group treated with cell receptor antibody, resulting in fewer plaques, while the outcomes for the HSPA1 antibody group were similar to those of the IgG control group (Fig. 2B), indicating that the HSPA1 antibody did not impact FMDV entry. These findings suggested that HSPA1 does not influence the entry stage of FMDV.

Next, we validated the role of HSPA1 in the RNA replication stage of FMDV. The key indicator of the virus RNA replication process was the production of viral negative-sense RNA (29). By manipulating HSPA1 expression in PK-15 cells through overexpression or knockdown, followed by infection at an MOI of 1, we collected samples at 1, 2, 4, and 6 hpi to measure viral RNA levels in both total RNA and negative-sense RNA. The findings indicated that overexpression of HSPA1 significantly reduced the viral RNA levels of both total RNA and negative-strand RNA (Fig. 2C and D) at 4 and 6 hpi, whereas knockdown of HSPA1 led to increased levels of total RNA (at 4 hpi) and negative-strand RNA (at 2, 4, and 6 hpi) compared to the NC group (Fig. 2E and F). This result revealed the inhibitory effect of HSPA1 on FMDV infection during the RNA replication stage.

Further investigation was conducted to assess the role of HSPA1 in the translation stage of FMDV using a dual-luciferase assay. FMDV translation was dependent on its IRES, located in the UTR at the 5′ end of the viral genome (24). Therefore, a dual-luciferase plasmid previously developed and stored in our laboratory (Fig. 2G) was employed. The results revealed that neither overexpression nor knockdown of HSPA1 alongside transfection of psiCHECK-FMDV influenced the translation efficiency of the FMDV 5′UTR (Fig. 2H and I). This suggested that variations in HSPA1 protein levels did not impact the viral translation process.

Collectively, these findings demonstrated that HSPA1 inhibited the RNA replication of FMDV.

### HSPA1 exerted a degradation effect on the 3D protein of FMDV

**Fig 3:**
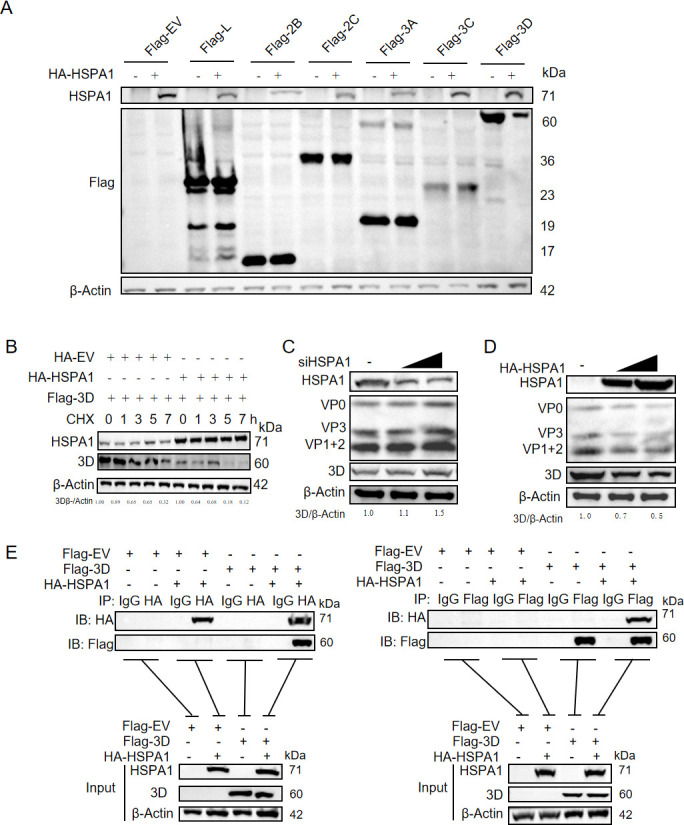
HSPA1 exerted a degradation effect on the 3D protein of FMDV. (**A**) PK-15 cells were transfected with HA-EV or HA-HSPA1 plasmids. After 24 hours, the cells were transfected with each FMDV non-structural protein plasmid or a Flag-tagged empty plasmid (Flag-EV). After 24 hours of transfection, samples were harvested for immunoblotting analysis. (**B**) PK-15 cells were transfected with either HA-EV or HA-HSPA1 plasmids. After 24 hours, the cells were transfected with Flag-3D plasmid. After another 24 hours, the cells were treated with CHX, 100 µg/mL, and subsequently harvested at the indicated time points. Immunoblotting analysis was then performed to assess the degradation effect of HSPA1 on viral 3D. Relative 3D protein levels were quantified using densitometry in Image J, with β-actin serving as a loading control for normalization. PK-15 cells were infected with FMDV at an MOI of 1, 24 hours post-HSPA1 knockdown (**C**) or overexpression (**D**). The samples were collected at 8 hpi. Immunoblotting analysis was then performed. Relative 3D protein levels were quantified using the same method as above. (**E**) Co-immunoprecipitation (Co-IP) analysis of HSPA1 and 3D was conducted. PK-15 cells were transfected with HA-HSPA1 and viral 3D plasmid. Cell lysates were immunoprecipitated with either IgG/HA or IgG/Flag antibodies, followed by immunoblotting with HA and Flag antibodies, respectively.

During the viral RNA replication stage, FMDV RNA replication occurred in the cytoplasm, involving the rearrangement and increase of intracellular membranes to form viral RC, which consisted of double-membrane vesicles (30, 31). Both the replication and translation of FMDV RNA took place within these complexes, with negative-sense RNA synthesis facilitated by the viral 3D polymerase. Various non-structural proteins of the virus participated in the viral RNA replication process (32–36). Therefore, we examined the effect of HSPA1 on these viral non-structural proteins. After co-overexpressing HSPA1 with plasmids encoding different FMDV non-structural proteins, the results demonstrated that overexpression of HSPA1 significantly inhibited the expression of the viral 3D protein (Fig. 3A). To further investigate this, we employed CHX, a protein synthesis inhibitor, to assess the impact of HSPA1 on the stability of the 3D protein. The findings indicated that in the presence of CHX, HSPA1 overexpression significantly reduced the stability of the 3D protein, promoting its degradation (Fig. 3B). To investigate the impact of HSPA1 on FMDV 3D protein during infection, cells were infected with FMDV at an MOI of 1, 24 hours post-HSPA1 knockdown or overexpression. The results revealed that, accompanied by changes in viral structural protein levels, 3D protein levels increased upon HSPA1 knockdown (Fig. 3C), while HSPA1 overexpression led to a marked decrease in 3D levels (Fig. 3D). These findings provide further evidence that HSPA1 plays a critical role in promoting the degradation of FMDV 3D protein. Additionally, immunoprecipitation assays revealed that HSPA1 is directly bound with the 3D polymerase (Fig. 3E). These results suggested that HSPA1 inhibited viral RNA replication by directly binding to the 3D polymerase and facilitating its degradation.

### HSPA1 directly bound to 3D through its peptide-binding domain

**Fig 4:**
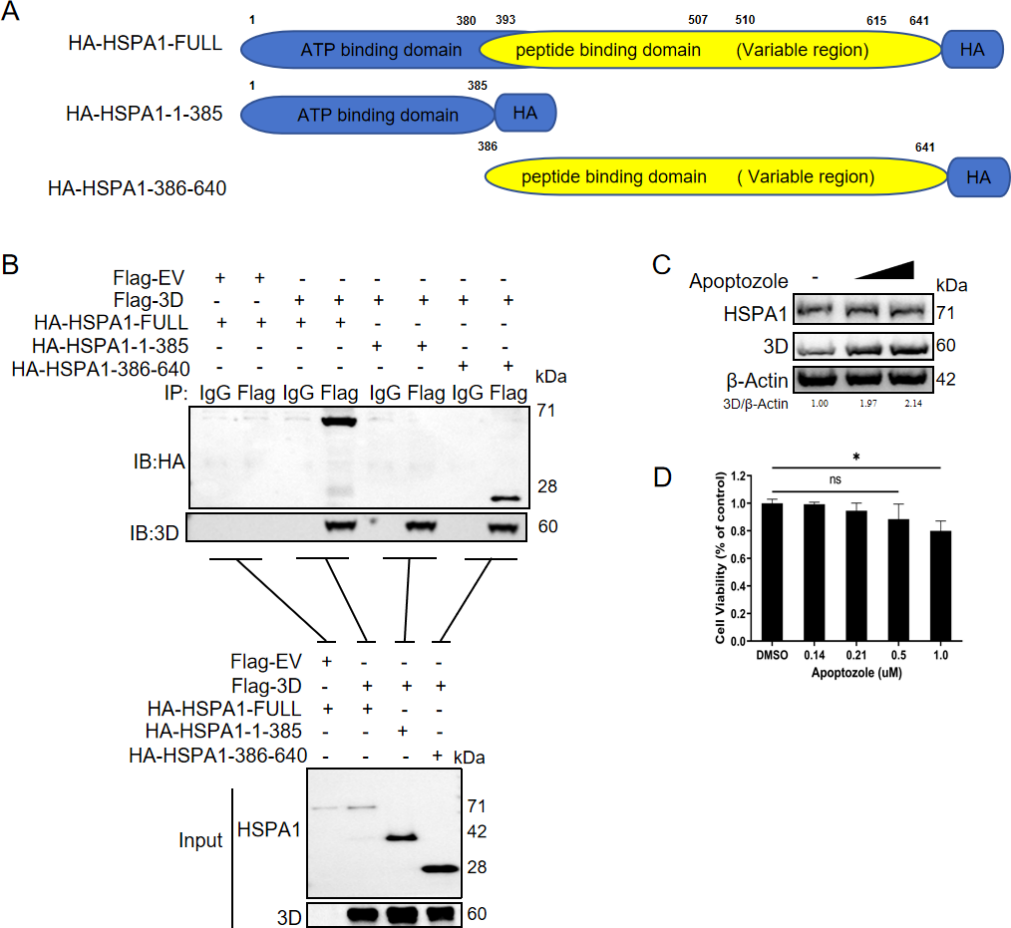
HSPA1 is directly bound to 3D through its peptide binding domain. (**A**) A schematic diagram illustrating the design of HSPA1 truncated versions. (**B**) Co-IP analysis of HSPA1 truncated versions and 3D. PK-15 cells were co-transfected with Flag-3D and HSPA1 truncation plasmids, and cell lysates were immunoprecipitated with IgG/Flag antibodies, followed by immunoblotting with HA and Flag antibodies. (**C**) PK-15 cells were treated with the ATPase function domain inhibitor of HSPA1 (Apoptozole) at concentrations of 0.14 µM, 0.21 µM of DMSO. After 24 hours, cells were transfected with Flag-3D plasmid. After another 24 hours, the relative protein level of 3D was assessed by immunoblotting assay. (**D**) The cell viability of Apoptozole was determined using the MTT assay. Data were presented as the mean ± standard deviation of three independent experiments. Statistical significance was indicated as follows: *, *P* < 0.05; ns, *P* > 0.05.

Structurally, HSPA1 was composed of an ATP-binding domain and a peptide-binding domain (37). To investigate which domain of HSPA1 connected with 3D, we constructed two plasmids with truncated HSPA1: HA-HSPA1-1-385, representing the ATP-binding domain, and HA-HSPA1-386-640, representing the peptide-binding domain (Fig. 4A). The two plasmids containing truncated HSPA1 were individually co-transfected with 3D plasmid into PK-15 cells, and samples were collected 24 hours post-transfection for immunoprecipitation assays. The finding indicated that the 3D protein was specifically bound to the peptide-binding domain of HSPA1 (Fig. 4B).

The mode of action of HSPA1 was delineated into two main steps. The peptide-binding domain of HSPA1 was specifically bound to the target protein. The substrate binding and release cycle of Hsp70 was regulated by its switch between an ATP-bound state and an ADP-bound state. Therefore, ATP binding and hydrolysis were crucial for its chaperone activity (37). After confirming that the viral 3D protein is connected with the peptide-binding domain, we further explored the involvement of the ATP-binding domain. Utilizing Apoptozole, a functional inhibitor of the ATP-binding domain of HSPA1, we observed that the degradation effect of HSPA1 on the 3D protein was inhibited (Fig. 4C). The cytotoxicity of Apoptozole in PK-15 cells was assessed before with an MTT assay, revealing minimal toxicity within the used dose range (Fig. 4D). These results collectively suggested that HSPA1 directly connected with the 3D protein through its peptide-binding domain, while its ATP-binding domain also played a crucial role in this degradation process, consistent with the known structure and function of HSPA1.

### HSPA1 facilitated the degradation of 3D via chaperone-mediated autophagy pathway

**Fig 5:**
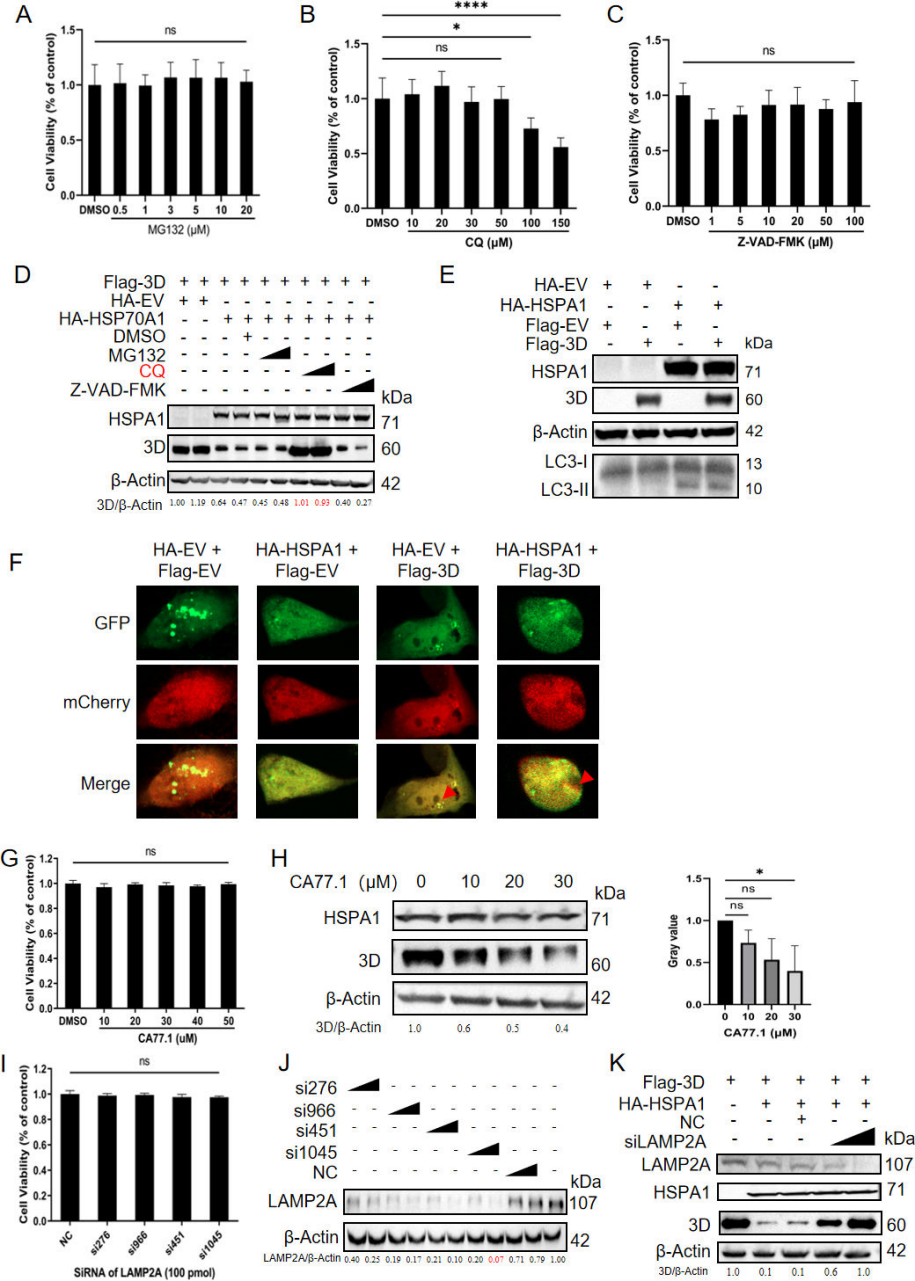
HSPA1 facilitated the degradation of 3D via the CMA pathway. The cell viability of MG132 (**A**), CQ (**B**), Z-VAD-FMK (**C**), CA77.1 (**G**), and siRNA targeting LAMP2A (**I**) was determined using the MTT assay. (**D**) PK-15 cells were co-transfected with HA-HSPA1 or HA-EV and the Flag-3D plasmid, and then maintained in the presence of DMSO, MG132 (2 or 20 µM), Z-VAD-FMK (10 or 50 µM), or CQ (20 or 50 µM). After 24 hours, the relative protein level of 3D was determined by immunoblotting analysis. (**E**) PK-15 cells were transfected with HA-HSPA1 plasmid. After 24 hours, Flag-3D or Flag-EV plasmids were transfected to these cells. After another 24 hours, the cells were harvested. Immunoblotting analysis was performed to examine the appearance of the LC3-II band and changes in the ratio of LC3-I/II. (**F**) Confocal microscopy analysis of macroautophagy activation. PK-15 cells were co-transfected with HA-HSPA1 or HA-EV and the Flag-3D or Flag-EV, alongside the pCMV-mCherry-GFP-LC3B, an LC3 plasmid fused with mCherry and GFP tags. Upon macroautophagy activation, LC3 would translocate into acidic lysosomes, resulting in GFP fluorescence quenching and the retention of mCherry red fluorescence. DAPI staining was omitted due to interference with mCherry fluorescence, as LC3 localization and autophagic activity are cytoplasmic processes. Individual red fluorescence images are marked with red triangles. (**H**) PK-15 cells were transfected with Flag-3D plasmid. After 24 hours, the cells were treated with CA77.1 at different concentrations of DMSO (the 0 mM group in the figure). After another 24 hours, the cells were harvested to perform immunoblotting analysis. Relative 3D protein levels were quantified using densitometry in Image J, with β-actin serving as a loading control for normalization. Statistical evaluation of grayscale values from multiple independent experiments was performed. Data were presented as the mean ± standard deviation of three independent experiments. Statistical significance was indicated as follows: *, *P* < 0.05; ns, *P* > 0.05. (**J**) The knockdown efficiency of siRNA targeting LAMP2A was verified. PK-15 cells were transfected with four groups of siRNAs targeting LAMP2A or NC. After 24 hours, the cells were harvested. Immunoblotting analysis was performed to determine the protein level of LMAP2A. (**K**) SiLAMP2A was first transfected into PK-15 cells. At 24 hours post-siRNA transfection, Flag-3D or Flag-EV were transfected. Subsequently, at 48 hours post-siRNA transfection, HA-HSPA1 or HA-EV were introduced. After another 24 hours, the cells were harvested to perform immunoblotting analysis. The relative 3D protein levels were quantified using densitometry in Image J, with β-actin serving as a loading control for normalization.

To further elucidate the specific degradation pathway employed by HSPA1 to degrade 3D, we used inhibitors targeting three protein degradation pathways: the proteasome inhibitor MG132, the autophagy inhibitor CQ, and the apoptosis inhibitor Z-VAD-FMK. The cytotoxicity of these inhibitors on PK-15 cells was assessed by MTT assays, showing no cytotoxic effects at the concentrations used in this study (Fig. 5A through C). After transfecting PK-15 cells with HA-HSPA1 for 24 hours, Flag-3D was transfected, followed by inhibitor treatment, and samples were collected after 24 hours. The results demonstrated that the degradation effect of HSPA1 on 3D was inhibited when cells were treated with the autophagy inhibitor (Fig. 5D).

Subsequently, we investigated whether HSPA1 degrades the 3D protein through macroautophagy. LC3, microtubule-associated protein 1 light chain 3, served as well-known as a marker of autophagosome membranes (38). During macroautophagy, the cytoplasmic form LC3-I would convert to the active, membrane-bound form LC3-II through cleavage and lipidation (39). The detection of LC3-II was used to monitor macroautophagy. Following co-transfection of HA-HSPA1 or HA-EV with either Flag-3D or Flag-EV, the results revealed that while HSPA1 overexpression alone induced the accumulation of LC3-II, the presence of Flag-3D did not affect the protein levels of LC3-I/II (Fig. 5E). To further explore the possibility that HSPA1 mediated 3D protein degradation via macroautophagy, we employed the pCMV-mCherry-GFP-LC3B plasmid (Fig. 5F). This plasmid encodes a dual-labeled LC3B fusion protein with red mCherry and green GFP fluorescent tags. During macroautophagy, autophagosomes fuse with lysosomes, where the acidic environment quenches GFP fluorescence. In contrast, mCherry retains its fluorescence under acidic conditions due to its exceptional stability. Thus, the mCherry-GFP-LC3B fusion protein allows for precise monitoring of autophagic flux (40). However, our repeated experiments consistently showed that DAPI staining led to the loss of mCherry fluorescence, which prompted us to omit nuclear staining in this study to preserve the integrity of mCherry signals. Using confocal microscopy, we observed that transfection with HA-EV and Flag-3D did not result in GFP quenching, indicating that macroautophagy was not induced. However, cells transfected with HA-HSPA1 exhibited mCherry fluorescence alone, suggesting active macroautophagy. In the HA-HSPA1 and Flag-3D co-transfection groups, red fluorescence was similarly observed, confirming the occurrence of macroautophagy. However, the number of autophagosomes (the red dots) did not change significantly. This finding aligned with the results presented in Fig. 5E.

Next, given the chaperone function of HSP70s, we explored whether HSPA1-mediated degradation of 3D occurs through the CMA pathway, a substrate-specific mode of lysosomal proteolysis (41). The CMA pathway selectively degraded proteins containing the KFERQ-like motif. This KFERQ-like motif needs to consist of one or two positively charged residues (K, R), one or two hydrophobic residues (I, L, V, F), one negatively charged residue (D, E), and a Q positioned at either side of the pentapeptide. Additionally, phosphorylation (Ser/Thr → E/D) or acetylation (Lys → Ac-Lys) can modify proteins to create functional CMA-recognition motifs, allowing them to be targeted for degradation. Using the KFERQ motif detection tool (KFERQ finder V0.8, https://rshine.einsteinmed.edu/) developed by Kirchner et al. (42), we identified two potential KFERQ motifs (_160_QTFLK_164_, _421_QEKLI_425_) in the 3D protein, suggesting its potential recognition by the CMA pathway. After transfecting Flag-3D, we treated cells with an activator of the CMA pathway, CA77.1, and observed changes in the level of the 3D protein. The cytotoxicity of CA77.1 in PK-15 cells was verified by an MTT assay, revealing negligible toxicity (Fig. 5G). The results showed a decrease in the 3D protein levels as CA77.1 concentrations continued to increase (Fig. 5H). Also, a grayscale analysis of protein bands was performed, followed by a statistical evaluation of grayscale values from multiple independent experiments. The results revealed a significant reduction in the intensity of the 3D protein band when the concentration of CA77.1 reached 30 µM, compared to the DMSO control group (0 µM, as shown in Fig. 5H). This finding indicated that the activation of the CMA pathway using CA77.1 effectively diminishes 3D protein levels.

Lysosomal receptor lysosomal-associated membrane protein 2 (LAMP2A) served as a receptor and channel for transporting cytosolic proteins during CMA and was used as a target to block the CMA pathway in research (43–46). To further confirm the HSPA1-mediated degradation of 3D protein occurred through the CMA pathway, we blocked the pathway by using siRNA to knock down LAMP2A. The cytotoxicity of these siRNAs in PK-15 cells was also verified by MTT assay and found to be negligible (Fig. 5I). As shown in Fig. 5J, si1045 effectively knocked down LAMP2A protein levels. For clarity, si1045 was referred to as siLAMP2A in the subsequent text. Upon LAMP2A knockdown using siLAMP2A, the degradation effect of overexpressed HSPA1 on the 3D protein was blocked, leading to an increase in 3D protein levels (Fig. 5K). Collectively, given that the activation or inhibition of the CMA pathway significantly altered the degradation of the 3D protein, while no notable changes in the LC3-I/II ratio, a hallmark of macroautophagy, were observed during this process, we focused our subsequent investigations on the potential role of HSPA1 in mediating 3D protein degradation via the CMA pathway.

### The key motif in the 3D degradation by the CMA pathway

**Fig 6:**
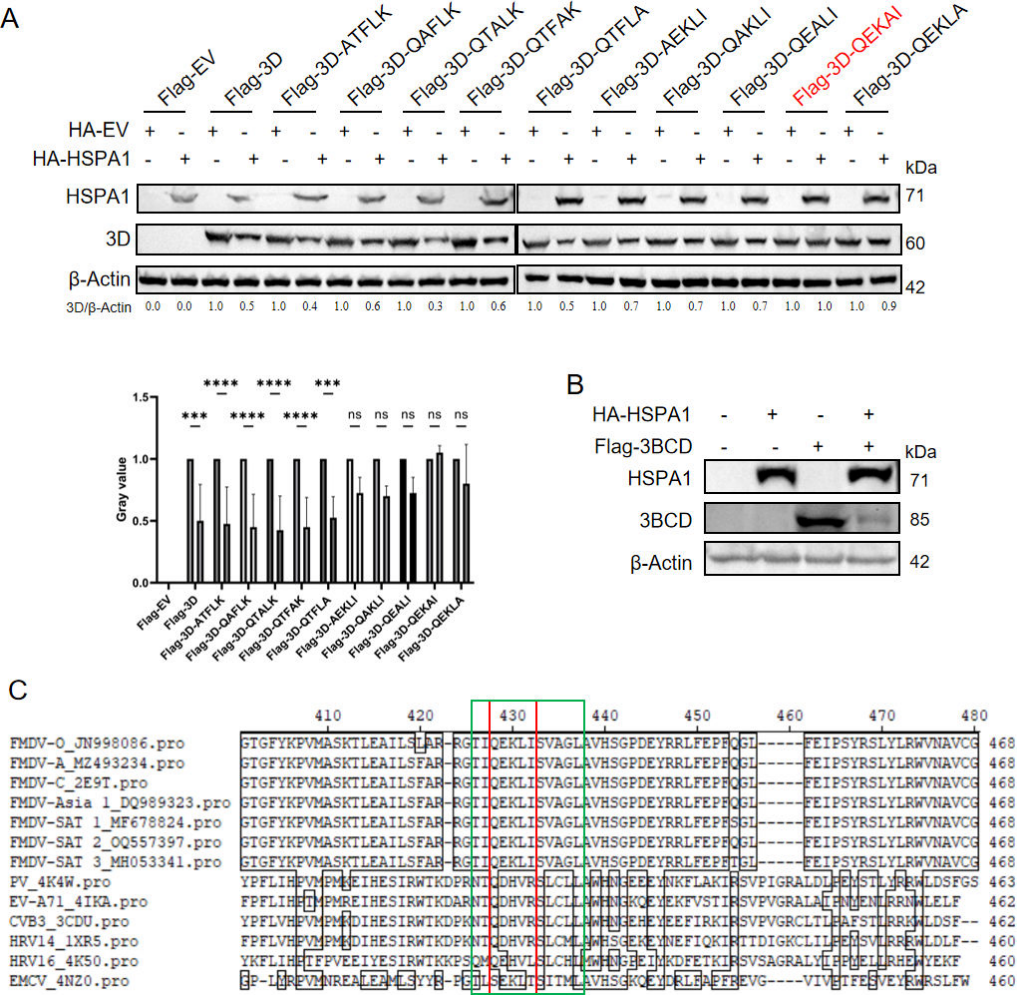
Blocking the CMA pathway promoted FMDV replication. (**A**) PK-15 cells were transfected with either HA-HSPA1 or HA-EV plasmids. After 24 hours, the cells were transfected with Flag-3D or Flag-EV or mutated 3D plasmids. After another 24 hours, the cells were harvested to perform immunoblotting analysis. The relative 3D protein levels were quantified using densitometry in Image J, with β-actin serving as a loading control for normalization. Statistical evaluation of grayscale values from multiple independent experiments was performed. Data were presented as the mean ± standard deviation of three independent experiments. Statistical significance was indicated as follows: ***, *P* < 0.001; ****, *P* < 0.0001; ns, *P* > 0.05. (**B**) PK-15 cells were transfected with Flag-3BCD or Flag-EV. After 24 hours, HA-HSPA1 or HA-EV plasmids were transfected to these cells. After another 24 hours, the cells were harvested to perform immunoblotting analysis. (**C**) Comparison of the amino acid sequences of 3D proteins from different members of the picornavirus family. The green box highlighted the conserved thumb α-helix structural feature in the 3D protein of picornaviruses, while the red box indicated the QEKLI motif in the FMDV 3D protein and its corresponding sequences in other picornaviruses. The amino acid sequences of 3D proteins from various picornavirus family members were obtained from GenBank (National Center for Biotechnology Information) or the Protein Data Bank (PDB). The accession numbers are as follows: FMDV type O, JN998086; FMDV type A, MZ493234; FMDV type Asia 1, DQ989323; FMDV type SAT 1, MF678824; FMDV type SAT 2, OQ557397; FMDV type SAT 3, MH053341. The PDB entries include FMDV type C, 2E9T; EV-A71, 4IKA; CVB3, 3CDU; PV, 4K4W; EMCV, 4NZ0; HRV14, 1XR5; HRV16, 4K50. These sequences were aligned using the Clustal W method in MegAlign 7.1.0, DNASTAR (USA).

As previously mentioned, CMA is a selective autophagy process that specifically recognizes and degrades proteins containing KFERQ-like motifs (41). The FMDV 3D protein contained two such motifs. Therefore, we further investigated which motif in the 3D protein is targeted by HSPA1 through the CMA pathway. To conduct the alanine scanning mutagenesis, we constructed 10 mutated 3D plasmids, each with alanine mutations at individual AA positions within both motifs. Cells were then co-transfected with either the HA-HSPA1 plasmid or HA-EV plasmid and the wild-type or mutant 3D plasmids, and the degradation effect of HSPA1 on the 3D protein was assessed (Fig. 6A). Protein band intensities were analyzed using grayscale measurements, and the resulting values were statistically evaluated across multiple independent experiments. The results demonstrated that mutagenesis of the _160_QTFLK_164_ motif resulted in a significant reduction in 3D protein levels upon HSPA1 overexpression. In contrast, mutations within the _421_QEKLI_425_ motif did not lead to significant changes in 3D protein levels. Notably, the _421_QEKAI_425_ mutant completely abolished HSPA1’s degradative effect. This result highlighted the critical role of the _421_QEKLI_425_ motif in facilitating HSPA1-dependent regulation. To further validate that HSPA1-mediated degradation of the 3D protein occurs through recognition of the _421_QEKLI_425_ motif, we utilized 3BCD, the precursor protein of 3D, which also contains the QEKLI motif. This approach aimed to confirm whether the presence of this specific motif in 3BCD similarly rendered it susceptible to HSPA1-dependent degradation. The results demonstrated a decrease in 3BCD protein levels with HSPA1 overexpression. This observation suggests that HSPA1 also mediates the degradation of 3BCD, consistent with its regulatory role observed for the 3D protein. These findings further reinforce the notion that HSPA1 targets proteins containing the QEKLI motif for degradation (Fig. 6B). Additionally, a comparison of the AA sequences of 3D proteins across different members of the picornavirus family revealed that only FMDV possesses the QEKLI sequence (the red box in Fig. 6C). The alignment of 3D sequences was conducted based on structural features that were reported by a previous study which identified this region as a conserved thumb α-helix structure among picornaviruses (the green box in Fig. 6C) (47). This relatively conserved AA region was described many times by fellow colleagues as NTQDHVRSLCLL in poliovirus (PV), EV-A71, and CVB3, NTQDHVRSLCML in human rhinovirus 14 (HRV14), QMQEHVLSLCHL in HRV16, TLSEKLTSITML in encephalomyocarditis virus (EMCV), and TIQEKLISVAGL in all 7 serotypes of FMDV (47–51). This finding indicated that, during HSPA1-mediated degradation of the 3D protein via the CMA pathway, the pathway specifically recognizes the _421_QEKLI_425_ motif.

### Blocking the CMA pathway promoted FMDV infection

**Fig 7:**
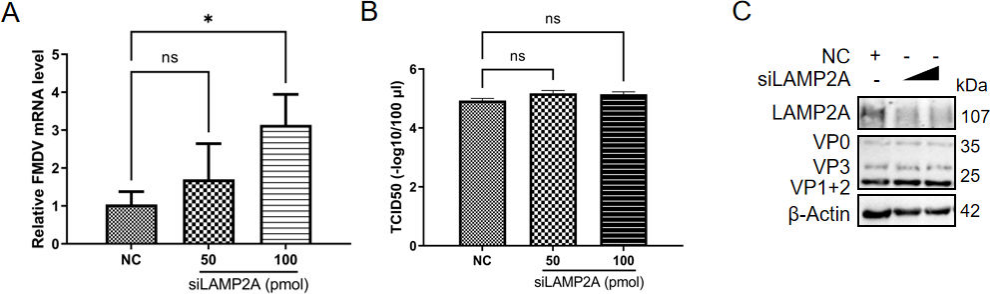
Blocking the CMA pathway promoted FMDV infection. PK-15 cells were transfected with either siRNA targeting LAMP2A (siLAMP2A) or NC. After 24 hours, PK-15 cells were infected with FMDV type O at an MOI of 1. Samples were collected at 8 hpi to assess relative FMDV RNA (**A**), titer (expressed in logarithmic form) (**B**), and viral protein levels (**C**). Data were presented as the mean ± standard deviation of three independent experiments. Statistical significance was indicated as follows: *, *P* < 0.05; ns, *P* > 0.05.

It is demonstrated in our study that HSPA1 degraded the viral 3D protein via the CMA pathway, thereby inhibiting viral RNA replication and ultimately suppressing FMDV infection. To further confirm whether blocking the CMA pathway would influence FMDV infection, we knocked down the LAMP2A protein to block the CMA pathway. Twenty-four hours later, cells were infected with FMDV, and samples were collected at 8 hpi. The results indicated that there was a significant increase in FMDV RNA levels (Fig. 7A), although there were no significant changes in the levels of viral proteins and titers (Fig. 7B and C). This suggested that blocking the CMA pathway promoted viral RNA replication, supporting the conclusion that the CMA pathway inhibits FMDV replication.

## DISCUSSION

HSPs are a class of functionally related proteins whose expression increases in response to elevated temperatures or other forms of cellular stress, including viral infection (52). They are distributed throughout various cellular compartments and are involved in protein refolding, translocation, complex assembly, and ATP-dependent protein transport. In addition to these functions, HSPs also mediate protein degradation and the regulation of multiple signaling pathways (53, 54). Among them, the relationship between the HSPA family and viral infections has been extensively studied. HSPAs have been widely reported to influence various stages of viral replication. For instance, HSPA5 promotes the attachment and internalization of the porcine epidemic diarrhea virus by interacting with the spike protein and the endo-/lysosomal pathway (55). The host Hsp70 network is essential for the entry, RNA replication, and virion production of the Dengue virus (56). Inhibiting HSP70 has been shown to block bovine viral diarrhea virus infection during the early stages and suppress its replication (57). Furthermore, HSP70 plays a functional role in both pre- and post-Zika virus infection processes, affecting viral entry, replication, and egress (58). However, HSPAs also function as negative regulators of infections for some other viruses. For example, ectopic HSP70 prevents the cytotoxic effects induced by the West Nile virus capsid (59). Additionally, HSP70 inhibits influenza virus replication by interacting with the PB1 and PB2 subunits of the viral ribonucleoprotein, thereby disrupting the binding of the viral polymerase to the viral RNA (60). In the picornavirus family, HSPAs have been implicated in the entire process of EV-A71 infection (16). Specifically, HSPA1 plays a crucial role in EV-A71 entry, IRES-mediated translation, and viral release. In this study, we first investigated the effect of HSPA1 on FMDV infection. The results indicated that HSPA1 exerts an inhibitory effect on FMDV infection. Subsequently, we explored the specific stages of the FMDV infection targeted by HSPA1.

Our study demonstrated that HSPA1 significantly inhibits the replication of FMDV. Overexpression of HSPA1 led to a marked reduction in viral RNA level, titer, and protein level (Fig. 1A through C), while its knockdown facilitated viral replication (Fig. 1F through H), indicating a critical role of HSPA1 in modulating viral proliferation. This finding aligns with previous studies, as mentioned above, reporting that molecular chaperones such as HSP70 family proteins can influence viral life cycles by affecting their infection process (59, 60). However, while inhibiting FMDV replication, HSPA1 was essential for EV-A71 entry, translation, assembly, and release (16). The divergent roles of HSPA1 in the replication processes of FMDV and EV-A71 are likely due to their distinct mechanisms of viral replication. For example, for FMDV, the interaction between integrins, particularly αvβ6, and the viral RGD motif is critical for its attachment and internalization (61). In contrast, EV-A71 relies primarily on SCARB2 as its principal receptor to facilitate entry into host cells (62). These differences in receptor utilization may underpin the contrasting effects of HSPA1 on the replication cycles of these two viruses.

**Fig 8:**
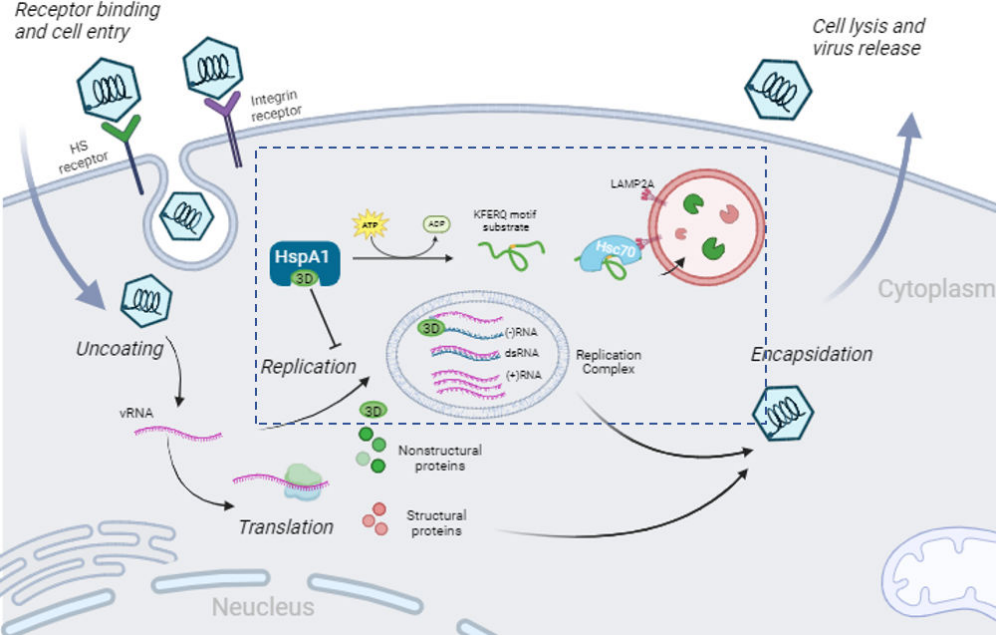
Schematic diagram of the hypothetical mechanism of HSPA1 inhibiting the RNA replication stage of FMDV. After FMDV enters the cells, its viral RNA is released into the cytoplasm and translated to viral proteins, including 3D. HSPA1 interacts with 3D and facilitates its degradation through the CMA pathway, thereby inhibiting the RNA polymerase activity of 3D during the RNA replication stage.

The lifecycle of picornaviruses begins with the virus binding to cell surface receptors (63, 64) (Fig. 8). FMDV attaches to integrin and HS receptors on the host cell surface, subsequently releasing its genomic RNA into the cytoplasm (25, 65, 66). The viral RNA serves as messenger RNA and is translated into the polyprotein (67, 68). The polyprotein is then processed into precursor proteins and, ultimately, mature proteins by the virus proteinases 2A and 3CD. Among these, the structural proteins later interact with newly synthesized positive-sense viral RNA to assemble new virions. The non-structural proteins, on the other hand, are directly involved in RNA replication. During replication, non-structural proteins catalyze the synthesis of viral negative-sense RNA within RCs formed through the rearrangement of intracellular membranes. This negative-sense RNA serves as a template for the synthesis of progeny positive-sense RNA. Therefore, the amount of viral negative-sense RNA reflects the progress of viral RNA replication. Once sufficient genomic RNA and structural proteins have been synthesized, the viral RNA is enclosed within capsids, forming progeny viruses, which are then released from the host cell. In this study, by verifying each stage of the virus replication process one by one, we found that HSPA1 inhibited the synthesis of FMDV negative-sense RNA, thereby suppressing viral RNA replication (Fig. 2A through I).

However, a comparison of Fig. 1A and F, as well as Fig. 2E and F, revealed distinct effects of HSPA1 knockdown on FMDV RNA levels. The negative-strand RNA levels were significantly increased in the HSPA1 knockdown group compared to the NC group at 2, 4, and 6 hpi. In contrast, while the total RNA levels also increased at 2, 4, and 6 hpi, the difference was only statistically significant at 4 hpi. We hypothesize that this phenomenon is due to the highly efficient RNA replication machinery of FMDV (68). The high efficiency of viral RNA replication ensures that multiple positive-strand RNAs can be derived from a single negative-strand RNA template, improving the efficiency of viral genome amplification. Similar findings have been reported in PV, another member of the picornavirus family (69). The study showed that during infection, the ratio of positive to negative strands ranges from approximately 40:1 to 70:1, peaking at 4 hpi. This pattern aligns with our results, where the most pronounced differences were observed at 4 hpi. At 6 hpi, the non-significant difference in total viral RNA levels may result from viral encapsidation, which leads to the packaging of a large amount of viral RNA and consequently reduces viral RNA replication.

Since the synthesis of viral negative-sense RNA relies on viral non-structural proteins, we further investigated the effect of HSPA1 on these proteins and discovered that HSPA1 degrades the viral 3D protein (Fig. 3A and B). The regulatory role of HSPA1 in 3D protein degradation was similarly observed during FMDV infection (Fig. 3C and D). This finding further supports the notion that HSPA1 facilitates the degradation of this crucial viral polymerase. In picornaviruses, the 3D protein serves as the RNA polymerase and directly participates in the synthesis of FMDV negative-sense RNA (47). This finding explains how HSPA1 negatively impacts the synthesis of viral negative-sense RNA, thereby inhibiting viral replication. Our observation that HSPA1 directly binds to 3D protein through its peptide-binding domain (Fig. 4B) underscores its specific interaction with this essential viral polymerase. Moreover, the involvement of the ATP-binding domain (Fig. 4C) highlights the energy-dependent nature of this process, which is consistent with HSPA1’s established role as a molecular chaperone (18).

Building on this, the question arises: which degradation pathway is utilized by HSPA1 to target 3D protein? By utilizing inhibitors of protein degradation pathways (Fig. 5D), we proposed that HSPA1 facilitated the degradation of 3D proteins via the autophagy pathway. Autophagy consists of three main pathways: macroautophagy, microautophagy, and CMA (40, 41). Macroautophagy has been widely reported to be involved in the replication of FMDV. FMDV infection induces ER stress and the unfolded protein response, triggering autophagy to restore cellular homeostasis (70). Sec62 interacts with LC3 to regulate ER stress and promote FMDV clearance via lysosomal fusion (71). However, FMDV exploits autophagy to suppress host defenses; VP1 inhibits innate immunity, and VP3 promotes autophagy to degrade HDAC8 through an AKT-MTOR-ATG5 pathway (72, 73). Interestingly, rapamycin-induced autophagy increases viral yield, while inhibiting autophagy reduces replication (74). These findings highlight the complexity of autophagy as both a defense mechanism and a viral replication tool. For microautophagy, no specific markers have been identified to date. Unlike macroautophagy, which involves independent membrane structures such as autophagosomes to encapsulate degraded products, microautophagy lacks such distinct features. The endosomal sorting complex required for transport, essential for microautophagy, is also crucial for macronucleophagy (75). Consequently, it cannot serve as a specific marker for microautophagy. This limitation hindered our ability to verify whether HSPA1 mediates the degradation of 3D through this pathway.

By analyzing LC3-II protein levels (Fig. 5E), a key marker of macroautophagy, and utilizing the pCMV-mCherry-GFP-LC3B plasmid to monitor the macroautophagic process (Fig. 5F), the findings suggested that the degradation of 3D protein is unlikely to occur primarily through the macroautophagy pathway, given that the LC3-I/II showed no significant changes. For Fig. 5F, when we conducted the experiment, after DAPI staining, the fluorescence signal from mCherry could not be observed under the confocal microscope. Since macroautophagy occurs in the cytoplasm and is independent of nuclear staining, we concluded that the absence of nuclear staining would not significantly impact the interpretation of our results. Therefore, DAPI staining was omitted to preserve the clarity of mCherry fluorescence. Through modulation of the CMA pathway using its activator CA77.1 or knockdown of its specific marker LAMP2A, we demonstrated that HSPA1 mediates the degradation of 3D protein mainly via the CMA pathway (Fig. 5H and K). These findings highlight a potential specificity of the CMA pathway in mediating 3D protein degradation. The lack of significant changes in the LC3-I/II ratio suggests that macroautophagy is not the primary mechanism involved in this process. Instead, the observed dependence on CMA modulation underscores the critical role of HSPA1 in targeting 3D protein for degradation. This distinction aligns with the established function of CMA in selectively degrading cytosolic proteins containing KFERQ-like motifs (41), further supporting the hypothesis that HSPA1 mediates 3D turnover mainly through a targeted, rather than bulk, autophagic pathway.

As CMA is highly selective, targeting proteins with KFERQ-like motifs, to pinpoint the critical motif for HSPA1-mediated degradation of the 3D protein through the CMA pathway, we conducted alanine scanning mutagenesis on the two KFERQ-like motifs in the 3D protein. This analysis identified _421_QEKLI_425_ as the crucial motif, which was essential for the 3D degradation mediated by HSPA1 via the CMA pathway (Fig. 6A). Also, the observed degradation of 3BCD, 3D precursor, in response to HSPA1 overexpression highlights the broader substrate specificity of HSPA1 toward proteins containing the QEKLI motif (Fig. 6B). This result aligns with the proposed mechanism where HSPA1 selectively recognizes and facilitates the degradation of target proteins through this critical sequence. As a selective protein degradation pathway, the CMA pathway targets proteins containing KFERQ-like pentapeptide motifs through the recognition by cytosolic HSC70 and its co-chaperones, subsequently directing them to lysosomes. LAMP2A then multimerizes to form translocation complexes, enabling the substrates to unfold and translocate across the lysosomal membrane with the aid of lysosomal HSC70. Based on the findings of this study, we hypothesize that HSPA1 unfolds the 3D protein via its chaperone function, exposing the KFERQ-like motif, which in turn facilitates the recognition and degradation of the target protein by the CMA pathway.

Structurally, the critical motif _421_QEKLI_425_ of 3D protein is located in helix α14 (49), with _423_KL_424_ facing inward, potentially rendering them inaccessible for direct recognition by HSC70. We speculate that, upon binding of the 3D protein to the peptide-binding domain of HSPA1, HSPA1 induces the unfolding of 3D, thereby exposing the entire _421_QEKLI_425_ sequence for recognition by HSC70, leading to its subsequent degradation via the CMA pathway. However, the conformational changes of the 3D protein during this process remain unexplored and require further experimental validation. Consequently, based on Fig. 6C, we propose that the inhibitory effect of HSPA1 on the 3D protein and viral replication may be specific to FMDV and may not extend to other picornavirus family members, aligning with previous research on HSPA1 and enteroviruses (16, 76, 77).

In the FMDV-infected cells, after the knockdown of LAMP2A, the level of viral RNA increased (Fig. 7A), but no significant changes were observed in viral protein or titer levels (Fig. 7B and C). During viral infection, the virus hijacks the host cell machinery, suppressing the translation of host proteins while producing large quantities of viral proteins (68). For the unchanged viral protein and titer levels, we speculate that, despite an increase in viral RNA, the cellular translation machinery for proteins remains unaffected. Consequently, blocking the CMA pathway promotes an increase in viral RNA, but the level of viral structural proteins does not change, indicating an inability to package additional progeny virus, which ultimately results in no significant change in viral titer.

In conclusion, this study demonstrates that HSPA1 upregulation inhibits FMDV infection. This inhibitory effect is mediated through the degradation of the viral RDRP, 3D protein, via the CMA pathway, thereby inhibiting viral RNA replication and ultimately suppressing viral infection (Fig. 8). Our results indicate that HSPA1 upregulation significantly enhances CMA of the viral RdRp. However, under physiological conditions, in FMDV-infected PK-15 cells in the presence of endogenous HSPA1 alone, CMA is involved in viral replication, though to a lesser extent. This research elucidates the impact and mechanism of HSPA1 on FMDV replication, identifying HSPA1 as a potential therapeutic target for the prevention and treatment of FMDV infection.

## Data Availability

The data sets used and/or analyzed during the current study are available from the corresponding author upon reasonable request.
